# Eligible Infants Included in Neonatal Clinical Trials and Reasons for Noninclusion: A Systematic Review

**DOI:** 10.1001/jamanetworkopen.2024.41372

**Published:** 2024-10-25

**Authors:** Henna Shaikh, Allison N. J. Lyle, Ellie Oslin, Megan M. Gray, Elliott Mark Weiss

**Affiliations:** 1 Department of Pediatrics, University of Washington School of Medicine, Seattle; 2 Department of Pediatrics, University of Louisville School of Medicine, Norton Children’s Medical Group-Neonatology, Louisville, Kentucky; 3 Treuman Katz Center for Pediatric Bioethics & Palliative Care, Seattle Children’s Research Institute, Seattle, Washington

## Abstract

**Importance:**

Results of clinical trials can only represent included participants, and many neonatal trials fail due to insufficient participation. Infants not included in research may differ from those included in meaningful ways, biasing the sample and limiting the generalizability of findings.

**Objective:**

To describe the proportion of eligible infants included in neonatal clinical trials and the reasons for noninclusion.

**Evidence Review:**

A systematic search of Cochrane CENTRAL was performed by retrieving articles meeting the following inclusion criteria: full-length, peer-reviewed articles describing clinical trial results in at least 20 human infants from US neonatal intensive care units, published in English, and added to Cochrane CENTRAL between 2017 and 2022. Retrieved articles were screened for inclusion by 2 independent researchers.

**Findings:**

In total 120 articles met inclusion criteria and 91 of these (75.8%) reported the number of infants eligible for participation, which totaled 26 854 in aggregate. Drawing from these, an aggregate of 11 924 eligible infants (44.4%) were included in reported results. Among all eligible infants, most reasons for noninclusion in results were classified as modifiable or potentially modifiable by the research team. Parents declining to participate (8004 infants [29.8%]) or never being approached (2507 infants [9.3%]) were the 2 predominant reasons for noninclusion. Other modifiable reasons included factors related to study logistics, such as failure to appropriately collect data on enrolled infants (859 of 26 854 infants [3.2%]) and other reasons (1907 of 26 854 infants [7.1%]), such as loss to follow-up or eligible participants that were unaccounted for. Nonmodifiable reasons, including clinical change or death, accounted for a small proportion of eligible infants who were not included (858 of 26 854 infants [3.2%]).

**Conclusions and Relevance:**

This systematic review of reporting on eligible infants included and not included in neonatal clinical trials highlights the need for improved documentation on the flow of eligible infants through neonatal clinical trials and may also inform recruitment expectations for trialists designing future protocols. Improved adherence to standardized reporting may clarify which potential participants are being missed, improving understanding of the generalizability of research findings. Furthermore, these findings suggest that future work to understand why parents decline to participate in neonatal research trials and why some are never approached about research may help increase overall participation.

## Key Points

**Question:**

What proportion of infants eligible for inclusion in neonatal clinical trials are not included, and what are the reasons for noninclusion?

**Findings:**

This systematic review of 120 studies reporting on US-based neonatal clinical trials found that many incompletely described inclusion of eligible neonates. In aggregate, 44.4% of eligible infants were included in trial results with key reasons for noninclusion described as potentially modifiable (eg, parents declining ([29.8%]) or modifiable (eg, parents never having been approached [9.3%]).

**Meaning:**

In this systematic review, more than one-half of eligible infants were not represented in trial results, with many reasons for noninclusion categorized as modifiable or potentially modifiable, representing possible targets to increase participation in future trials.

## Teaser

This systematic review assesses participant flow in neonatal clinical trials and reasons for noninclusion of eligible participants.

## Introduction

Clinical trials provide foundational evidence for medical therapies,^1^ yet their findings only reflect those who participate.^2^ Trial recruitment is particularly challenging in neonatal clinical trials, where parents are asked to provide surrogate consent for newborns during a time of substantial stress, and sometimes involve time-sensitive or investigational therapies.^3,4,5,6,7,8,9,10^ Several neonatal trials have been underpowered,^11,12^ have terminated early,^13,14,15,16,17,18^ or have failed to represent the general clinical population.

Increasing representativeness of neonatal trials requires understanding why eligible patients are not included. The Consolidated Statement of Reporting on Trials (CONSORT) presents guidelines for describing participant flow through trials, including how many patients are screened, found to be eligible, allocated to an intervention, and included in data analysis, as well as reasons that eligible patients are not included.^8,19^ These data can facilitate efforts to increase trial participation, which can reduce bias in results and improve the generalizability and representativeness of available evidence.^2^

Participant flow reporting has improved since the CONSORT guidelines were published,^20,21^ but reviews suggest that reporting remains incomplete, even among journals endorsing these guidelines.^2,22,23,24^ To our knowledge, no assessment of adherence to CONSORT guidelines exists for reporting in neonatal trials. Therefore, our objective was to review published neonatal trials to (1) calculate aggregate participant flow through trials and reasons for noninclusion of eligible participants and (2) assess adherence to CONSORT guidelines on participant flow reporting. We posit that our findings can inform efforts to improve recruitment of infants in neonatal clinical trials.

## Methods

The methods for this systematic review have been previously described.^25^ Our methods adhered to the Preferred Reporting Items for Systematic Reviews and Meta-Analyses (PRISMA) reporting guideline. We completed all items in the PRISMA checklist, including identifying a target database to search, delineating a comprehensive search strategy, thoughtfully crafting inclusion and exclusion criteria for studies, and defining a rigorous process for screening studies and identifying those eligible for inclusion. We also devised a flow diagram of screened and included manuscripts.^26^ Our planned methodology was published on PROSPERO.

We searched Cochrane CENTRAL in February 2022 using the search strategy previously described.^25^ We retrieved full-length, peer-reviewed articles describing trials including at least 20 infants in the US, published in English, and added to Cochrane CENTRAL between 2017 and 2021. We limited our search to US clinical trials because of the difference in recruitment practices and enrollment rates across countries. Unfortunately, in our preparatory work for this review, we found that for studies with both US and non-US sites, it was not possible to extract site-specific or country-specific relevant information (eg, reasons for noninclusion among eligible US infants only). Therefore, we limited our review to clinical trials that only enrolled US infants. Two researchers (H.S., A.L., or E.O.) screened retrieved articles. Disagreement on which articles met inclusion criteria was settled by group consensus. To avoid double-counting trial participants, only the first published manuscript from a clinical trial was included.

Data was extracted into a University of Washington-based REDCap^27,28^ database that reflected variables from CONSORT guidelines and was refined based on 4 research team members’ (H.S., A.L., M.G., and E.M.W.) pilot review of a sample of included articles. Variables extracted from each article included study characteristics and participant flow information, including numbers of patients screened and eligible for participation, allocated to an intervention, and included in the reported results of the primary outcome, along with reasons that eligible infants were not included in reported results. Given frequent inconsistencies and lack of clarity in trial reporting, at least 3 researchers (including H.S., A.L., E.O., and E.M.W.) met synchronously to categorize reasons for noninclusion. Reasons that could be changed by the research teams’ actions, such as increasing availability on nights and weekends, were categorized as modifiable. Reasons that could clearly not be changed, such as patient death, were categorized as nonmodifiable. Reasons that did not fit in either of those categories were labeled as potentially modifiable.

Our primary objective was to identify the number of infants eligible for participation in trials who were not included in reported analyses and the reasons for their noninclusion. Secondary objectives were to describe adherence to CONSORT guidelines on participant flow through research trials and characteristics of trials with higher and lower inclusion rates of eligible infants in reported analyses.

## Results

### Included Trials

**Figure 1.**
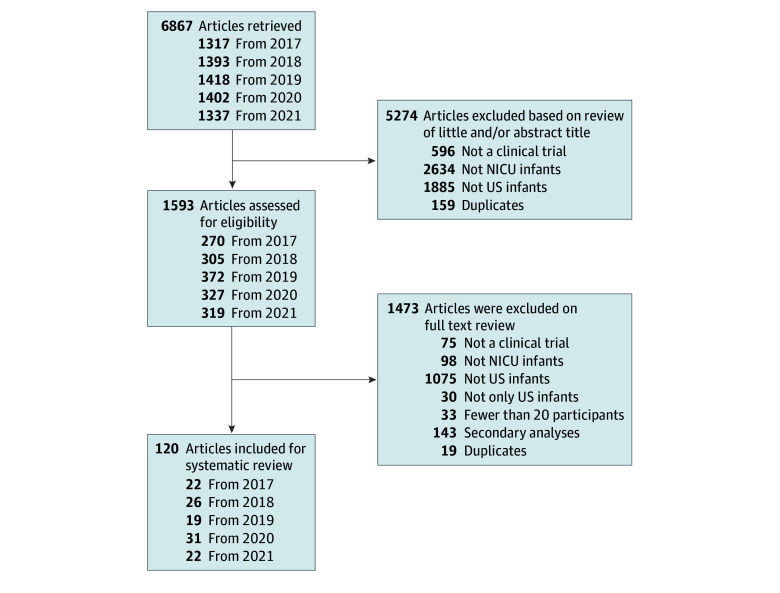
Diagram of Retrieved, Screened, and Included Articles NICU indicates neonatal intensive care unit.

We identified 120 publications that met inclusion criteria.^29,30,31,32,33,34,35,36,37,38,39,40,41,42,43,44,45,46,47,48,49,50,51,52,53,54,55,56,57,58,59,60,61,62,63,64,65,66,67,68,69,70,71,72,73,74,75,76,77,78,79,80,81,82,83,84,85,86,87,88,89,90,91,92,93,94,95,96,97,98,99,100,101,102,103,104,105,106,107,108,109,110,111,112,113,114,115,116,117,118,119,120,121,122,123,124,125,126,127,128,129,130,131,132,133,134,135,136,137,138,139,140,141,142,143,144,145,146,147,148^ A diagram of retrieved, screened, and included articles is shown in Figure 1. Comprehensive details of included studies are provided in the eTable in Supplement 1. Included articles were made available electronically between 2016 and 2021 and were published between 2017 and 2023 in 50 distinct journals. The most commonly included journals were the *Journal of Perinatology* (18 studies), *The Journal of Pediatrics* (17 studies), *Pediatric Research* (7 studies), *American Journal of Perinatology* (5 studies), *JAMA* (4 studies), *JAMA Pediatrics* (4 studies), *Journal of Parenteral and Enteral Nutrition* (4 studies), and *PLOS One* (4 studies). Trials took place in 38 distinct US states and the District of Columbia. Most trials enrolled infants postnatally only, although 16 enrolled infants prenatally in addition or exclusively,^29,30,31,37,41,57,86,88,112,117,121,126,128,134,144,145^ and 22 did not clearly state whether prenatal enrollment was possible.^43,46,52,54,55,60,63,90,93,99,103,109,113,123,124,130,133,136,140,142,147^ Eighteen of the included studies ended prior to reaching target enrollment.^30,32,40,45,46,50,61,70,78,86,91,114,121,122,127,134,140,145^ Among included articles, 93 reported the duration over which infants were enrolled, which ranged from 3 to 87 months (median [IQR] duration, 27 [18-42] months).^30,31,33,35,36,37,38,39,40,41,42,43,45,46,47,48,50,52,54,55,56,57,58,60,61,62,64,65,66,67,69,70,71,72,73,74,75,76,77,78,80,81,82,84,86,87,88,89,90,91,92,93,95,99,102,103,104,106,107,108,109,110,111,112,114,115,117,118,119,120,121,122,123,125,126,127,128,130,131,132,133,134,135,137,138,140,141,142,143,144,146,148^ All but one^43^ of the included articles reported the number of study sites; most (83 of 119 studies [70%]) described a single study site,^29,30,31,33,34,35,36,38,40,42,45,46,47,48,49,50,51,58,61,62,63,65,68,71,72,73,74,75,76,77,79,80,82,83,86,88,90,93,94,95,97,98,100,101,102,103,104,105,106,107,108,109,112,113,115,116,117,119,121,122,123,124,125,126,127,128,129,131,133,134,136,137,138,139,140,141,142,143,145,146,147^ and the remaining articles included up to 22 sites.^32,37,39,41,44,52,53,54,55,56,60,64,66,67,69,70,78,81,84,85,87,89,91,92,96,99,110,111,114,118,120,130,132,135,144,148^

### Adherence to CONSORT Guidelines on Participant Flow Reporting

Of the 120 included articles, 66 (55.0%) provided complete reporting on numbers of infants screened, eligible, allocated to an intervention, and included in the analysis, along with reasons for noninclusion of all eligible infants.^30,31,32,33,35,36,37,38,40,41,44,45,46,47,48,53,55,57,58,59,61,62,64,67,69,70,73,74,75,76,77,78,80,81,84,85,87,88,96,102,105,110,111,112,114,115,118,119,121,122,123,124,127,128,129,131,133,134,135,136,137,138,140,142,145,148^ All included articles presented numbers of infants allocated to the intervention and included in the primary outcome analysis. However, only 91 articles (75.8%) reported the number of infants eligible for inclusion,^29,30,31,32,33,35,36,37,38,39,40,41,44,45,46,47,48,51,53,54,55,56,57,58,59,60,61,62,63,64,65,67,69,70,72,73,74,75,78,80,81,82,84,85,86,87,88,89,90,91,92,96,97,100,102,103,105,106,107,109,110,111,112,114,115,116,118,119,121,122,123,124,126,127,128,129,131,133,134,135,136,137,138,139,140,141,142,144,145,148^ and only 74 (61.7%) reported the number screened for eligibility.^29,30,31,32,33,35,36,37,38,40,41,44,45,46,47,48,53,55,56,57,58,59,60,61,62,64,67,69,70,73,74,75,77,78,80,81,84,85,87,88,91,92,96,97,102,103,105,110,111,112,114,115,118,119,121,122,123,124,125,127,128,129,131,133,134,135,136,137,138,140,141,142,145,148^ Of the 91 articles that reported the number of infants eligible for inclusion, 12 (13.2%) failed to completely account for reasons that eligible infants were not included in the primary outcome analysis.^29,43,54,56,60,66,89,92,97,103,130,141^ In addition to numerical reporting, CONSORT guidelines recommend including a participant flow diagram in manuscripts; of all articles, 92 (76.6%) included a participant flow diagram,^29,30,31,32,35,36,37,38,39,40,41,44,45,46,47,48,51,52,53,54,55,56,57,58,59,61,62,63,64,65,67,69,70,72,73,74,75,76,77,78,81,82,83,84,86,87,88,89,90,91,92,96,97,98,99,102,103,104,105,106,107,109,110,111,112,114,115,116,117,118,119,121,122,123,124,126,127,128,129,131,133,134,135,136,137,138,140,141,142,144,145,148^ although the degree of completeness varied.

### Inclusion and Noninclusion of Eligible Participants

The 91 trials in this review that reported the number of eligible infants represented a total of 26 854 potential participants.^29,30,31,32,33,35,36,37,38,39,40,41,44,45,46,47,48,51,53,54,55,56,57,58,60,61,62,63,64,65,67,69,70,72,73,74,75,77,78,80,81,82,84,85,86,87,88,89,90,91,92,96,97,100,102,103,105,106,107,109,110,111,112,114,115,116,118,119,121,122,123,124,126,127,128,129,131,133,134,135,136,137,138,139,140,141,142,144,145,148^ In aggregate, 11 924 (44.4%) of these infants were included in reported results.

**Figure 2.**
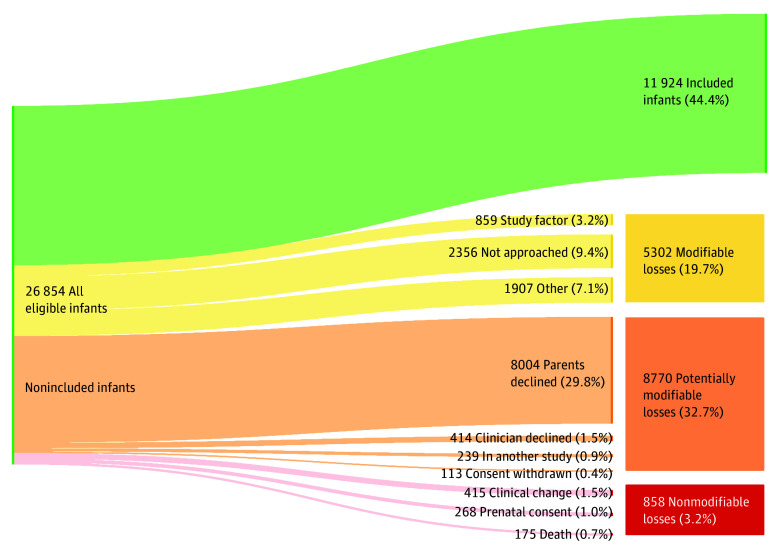
Aggregate Flow of Infants Eligible for Neonatal Clinical Trials Eligible infants were aggregated from 91 trials.^29,30,31,32,33,35,36,37,38,39,40,41,44,45,46,47,48,51,53,54,55,56,57,58,60,61,62,63,64,65,67,69,70,72,73,74,75,77,78,80,81,82,84,85,86,87,88,89,90,91,92,96,97,100,102,103,105,106,107,109,110,111,112,114,115,116,118,119,121,122,123,124,126,127,128,129,131,133,134,135,136,137,138,139,140,141,142,144,145,148^ Reasons for noninclusion were based on consensus categorization among study team members, with each category mutually exclusive. There was a discrepancy in reported numbers of participants who were and were not included such that the summed total of reported eligible infants was 23 fewer than the summed total of reasons provided for noninclusion. This figure incorporates these 23 additional infants into the all eligible participants category.

Articles described a plethora of reasons for not including eligible infants in reported results; a complete account of these reasons is provided in the eTable in Supplement 1. Through consensus, the research team grouped reasons for noninclusion into 10 categories, each of which was subsequently classified as modifiable, potentially modifiable, or nonmodifiable (Table 1). Figure 2 demonstrates the aggregate flow of all eligible infants included in this review through stages of trial participation. This figure shows proportions of eligible infants included in trial results and those not included for various reasons. Most reasons for noninclusion fell into categories that were classified as modifiable (5302 of 26 854 infants [19.7%]) or potentially modifiable (8770 of 26 854 infants [32.7%]). Parents declining to participate (8004 of 26 854 infants [29.8%]), classified as potentially modifiable, or never being approached (2507 of 26 854 infants [9.3%]), classified as modifiable, were the 2 predominant reasons for noninclusion. Other modifiable reasons included factors related to study logistics, such as failure to appropriately collect data on enrolled infants (859 of 26 854 infants [3.2%]) and other reasons (1907 of 26 854 infants [7.1%]), such as loss to follow-up or eligible participants that were unaccounted for. Nonmodifiable reasons accounted for 858 of 26 854 (3.2%) eligible infants not included in results.

**Table 1.** Definitions of Reasons for Noninclusion of Eligible Participants in Trial Results

Reason by ability to modify	Consensus definition and examples
Modifiable	
Study factor	Issues with the study design, equipment, team, or data collection, such as research team unavailable to recruit participant, problem with consent (eg, incomplete consent), enrolled in error, inadequate communication between research and clinical teams (eg, research team not notified about a potentially eligible participant or clinical team unaware that a patient is a research participant), unable to obtain baseline evaluation (eg, laboratory testing) to determine eligibility within window, participant received a therapy that precluded eligibility, protocol deviation (eg, participant did not receive correct intervention), equipment malfunction or user error of equipment, faulty or incomplete data collection, or study design issue that led to dropping participants after enrollment
Not approached	A specific study factor whereby no contact was made between the study team and the patient’s family with specific language such as “not approached,” “missed,” “unable to consent,” “parent/guardian unavailable,” “consent not requested,” or “outside window” for eligibility including because of shortened window due to emergent or precipitous delivery
Other	Other listed as the reason for noninclusion or eligible participants not included in results without any explanation, loss to follow-up, consenting party spoke a language other than English; reasons listed as combinations of other categories were listed here to avoid falsely increasing or decreasing numbers in other categories, concerns about the parents’ ability to consent (eg, related to age <18 y or clinical status), infant placed for adoption, or reasons that were vague or difficult to interpret such as “not expected to be available for follow-up,” “non-qualifying exclusion,” and “consented but not enrolled”
Potentially modifiable	
Parent declined	Parents declined or did not provide a response. Some studies gave a combined reason of “declined/not approached,” which was included here
Clinician declined	The clinician caring for the infant declined
Participating in another study	Infant was eligible but was participating in another study that precluded inclusion in the target study
Consent withdrawn	Consent withdrawn either before or after the intervention by the parent or the clinician
Nonmodifiable	
Clinical reason	Participant became too ill or was no longer eligible due to a change in clinical status; transfer to another facility was also included here
Prenatal consent	Consent was obtained prenatally; however, the participant was no longer eligible at the time of allocation to the intervention
Death	Participant died prior to outcome ascertainment

**Figure 3.**
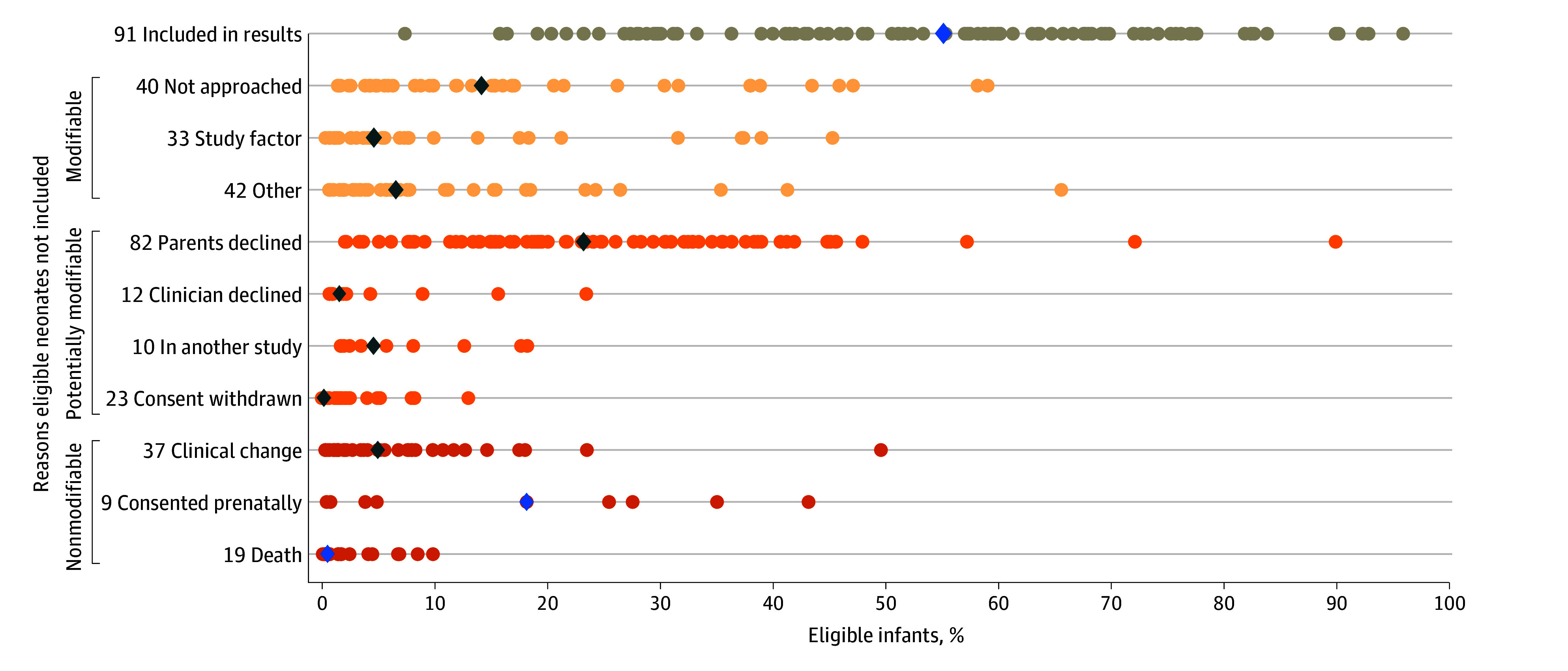
Variability in Proportions of Eligible Infants Included in Results and Not Included for Various Reasons Among Individual Trials The numbers on the y axis represent the number of trials for each reason for noninclusion among the 91 of 120 trials that included the number of infants eligible for participation. Each circle represents the proportion of eligible infants with a given outcome in a single study. Diamonds represent the median proportion of eligible infants with each outcome.

As shown in Figure 3, there was a great deal of variation among individual trials in the proportions of eligible infants included in reported results and not included for various reasons. The proportion of eligible infants included in reported results varied from 7.4% to 95.5%. Figure 3 shows that 5 trials ^44,102,107,133,134^ included a remarkably high proportion of eligible infants (>85%). Three of these trials evaluated relatively innocuous interventions (the Bedwell et al^136^ study on methods for warming human milk, the Kraft et al^46^ study on sublingual buprenorphine vs oral morphine for treatment of neonatal opioid withdrawal syndrome, and the Angeles et al^104^ study on pain management during routine neonatal intensive care unit (NICU) procedures with oral dextrose compared with facilitated tucking). However, 2 of these trials also investigated more intensive interventions, including the Pourmoghadam et al^109^ study of drain placement during cardiopulmonary bypass and the Ramanathan et al^135^ comparison of synthetic with porcine surfactant. Figure 3 also highlights one study with remarkably low inclusion of eligible infants, the study by Ceyhan-Birsoy et al^89^ that evaluated the clinical utility of genomic sequencing. This genomic sequencing study had a remarkably high proportion of parents who declined to participate (89.7%). In contrast, the study with the lowest proportion (2.0%) of parents who declined to participate was the Mu et al study^144^ comparing drawing admission laboratory testing from the umbilical cord with drawing from the infant.

### Participant Accrual Rate and Factors Associated With Inclusion of Eligible Infants

To inform trialists’ expectations on participant enrollment, we calculated a participant accrual rate based on the number of participants included in reported results divided by the number of months the study enrolled over and the number of study sites included. Among the 92 articles that reported all of these data points, the median (IQR) participant accrual rate was 1.7 (0.8-8.3) participants per study site per month.^31,33,35,36,37,38,39,41,42,45,46,47,48,50,52,54,55,56,57,58,60,61,62,64,65,66,67,69,70,71,72,73,74,75,76,77,78,80,81,82,84,86,87,88,89,90,91,92,93,95,96,99,102,103,104,106,107,108,109,110,111,112,114,115,117,118,119,120,121,122,123,125,126,127,128,130,131,132,133,134,135,137,138,140,141,142,143,144,146,148^

We also performed an exploratory analysis to describe how the proportion of eligible infants included in analyses varied according to study characteristics (Table 2). Statistical testing was not performed due to the small number of total studies; however, we reported trends in variation in enrollment rates according to study characteristics. Studies with prenatal enrollment tended to have a greater median (IQR) proportion of eligible participants included in the analysis compared with those with postnatal enrollment only (59.2% [28.1%-69.3%] vs 51.0% [36.2%-67.4%]). Studies relating to genetics had the lowest median (IQR) proportion of eligible participants included in the results (31.1% [7.4%-58.6%]), followed by studies of delivery room management (38.9% [28.1%-69.3%]), then general NICU care (51.5% [39.9%-63.5%)]), and finally, surgical studies having the greatest median (IQR) proportion of eligible participants included in results ( 67.4% [57.4%-75.5%]). There were approximately equal median (IQR) proportions of eligible participants who were included among studies funded by the National Institutes of Health or other governmental institution (51.0% [36.2%-67.5%]), a hospital (51.0% [31.4%-64.6%]), or a private organization (49.6% [28.9%-79.3%]), while there was a higher median (IQR) proportion among those funded by industry (57.1% [47.8%-60.0%]) or with no funding (65.6% [46.7%-74.7%]). Studies conducted at more than 5 sites had a lower median (IQR) proportion of included eligible participants (41.9% [29.7%-59.2%]) compared with those at 1 site (57.1% [36.2%-67.4%]) or 2 to 5 sites (56.3% [43.0%-75.3%]). There did not seem to be great differences in the proportion of included participants based on enrollment duration.

**Table 2.** Proportion of Eligible Infants Included in Results According to Study Characteristics

Study characteristics^a^	Studies, No.	Participants included in analysis vs eligible participants, median (IQR), %
Enrollment timing		
Prenatal	15^29,30,31,37,41,57,86,88,112,121,126,128,134,144,145^	59.2 (28.1-69.3)
Postnatal	62^32,35,36,38,39,40,44,45,47,48,51,53,56,58,59,61,62,64,65,67,69,70,72,73,74,75,77,78,80,81,82,84,85,87,89,91,92,96,97,100,102,104,105,106,107,110,111,114,115,116,118,119,122,127,129,131,135,137,138,139,141,148^	51.0 (36.2-67.4)
Study type		
General neonatal intensive care unit	67^32,35,37,39,40,41,44,45,46,47,48,51,53,55,56,58,59,61,62,63,64,65,67,69,70,73,74,75,78,80,81,82,84,85,90,91,92,96,97,102,103,104,105,107,111,114,115,116,118,119,122,123,124,127,128,129,131,133,135,136,137,138,139,141,142,144,148^	51.5 (39.9-63.5)
Delivery room	9^29,31,57,72,86,88,121,126,145^	38.9 (28.1-69.3)
Surgery	11^30,36,54,60,77,87,100,106,109,112,134^	67.4 (57.4-75.5)
Genetics	3^38,89,140^	31.1 (7.4-58.6)
Funding source^b^		
National Institutes of Health or government	45^32,36,37,38,40,44,45,46,54,55,56,59,62,63,64,65,67,69,70,72,75,77,81,87,88,89,92,96,103,105,106,107,110,114,115,122,124,127,128,129,137,139,140,141,148^	51.0 (36.2-67.5)
Hospital	15^32,38,45,58,59,75,77,82,85,102,109,123,124,131,138^	51.0 (31.4-64.6)
Industry	13^31,41,60,62,84,86,88,100,102,111,118,135,142^	57.1 (47.8-60.0)
Private organization	8^38,39,85,103,107,109,124,149^	49.6 (28.9-79.3)
No funding	4^47,97,112,144^	65.6 (46.7-74.7)
Sites, No.		
1	61^29,30,31,35,36,38,40,45,46,47,48,51,57,58,59,61,62,63,65,72,73,74,75,77,80,82,86,88,90,97,100,102,103,104,105,106,107,109,112,115,116,119,121,122,123,124,126,127,128,129,131,133,134,136,137,138,139,140,141,142,145^	57.1 (36.2-67.4)
2-5	16^32,39,44,53,54,56,60,69,78,85,89,92,110,111,118,144^	56.3 (43.0-75.3)
>5	14^37,41,55,64,67,70,81,84,87,91,96,114,135,148^	41.9 (29.7-59.2)
Enrollment duration, mo^c^		
≤18	21^31,35,38,41,45,47,48,56,62,70,72,75,78,88,103,107,110,115,118,121,148^	51.0 (36.2-67.8)
19-27	16^30,36,39,60,74,89,96,109,114,128,133,135,137,138,140,142^	58.0 (28.6-67.9)
28-42	16^37,58,61,65,69,73,77,82,84,92,102,106,119,123,126,141^	58.7 (44.2-67.7)
≥43	21^40,46,54,55,57,64,67,80,81,86,87,90,91,104,111,112,122,127,131,134,144^	63.3 (41.8-74.0)

^a^ Trials with missing information for each respective study characteristic were excluded, including 14 studies^46,54,55,60,63,90,103,109,123,124,133,136,140,142^ with missing data on prenatal vs postnatal enrollment, 23 studies^29,30,35,48,51,53,57,61,73,74,78,80,90,91,116,119,121,122,126,133,134,136,145^ with missing data on funding source, and 17 studies^29,44,51,53,59,63,85,97,100,105,116,124,129,136,139,145,149^ with missing data on enrollment duration. One study^110^ was excluded from the study type analysis because it fell into multiple categories.

^b^ Funding source categorizations were not mutually exclusive.

^c^ Enrollment duration categories were approximately based on quartiles.

## Discussion

This systematic review assessed the proportion of infants eligible for participation in neonatal clinical trials who were included in reported results and broadly categorized reasons for noninclusion of eligible infants who were not included. There are 3 key implications of our findings. First, there was wide heterogeneity in authors’ reporting of participant flow through trials, especially regarding reasons for noninclusion of eligible participants; this may limit clinician’s abilities to interpret trial findings and make comparisons across trials. Second, despite heterogeneity in reporting, it was clear that most reasons for noninclusion of eligible infants were modifiable or potentially modifiable, highlighting areas that could be targeted to increase recruitment. Third, overall participation of eligible infants was low, with fewer than one-half of aggregated eligible infants included in reported trial results. This finding can inform realistic expectations for trialists and funders designing and evaluating studies. Each of these points is discussed in turn below.

### Inadequate Adherence to CONSORT Reporting Guidelines

The CONSORT guidelines were first published in 1996^150^ and updated in 2001^151^ and 2010^19^ with the goal of improving the quality of reporting on clinical trials. CONSORT guidelines provide a 25-item reporting checklist and a templated participant flow diagram intended to guide reporting of patients included and excluded in trials, including reasons for exclusions of eligible patients. Previous reviews have found associations of adopting CONSORT with improved quality in trial reporting^20,21,23,152,153,154^; however, reporting remains incomplete.

Our review found that only 76.6% of included articles presented a participant flow diagram, and only 55.0% reported the numbers of patients screened for participation, eligible for participation, allocated to an intervention, and included in results along with reasons for noninclusion of eligible patients. Our findings are similar to reviews of adult oncology trials^155^ and adult and pediatric trials published in high impact journals,^156^ which both found that 52% of included articles reported the number of patients screened for eligibility. Additional reviews including trials from adult and pediatric populations found that 43% to 79% of articles presented a participant flow diagram.^155^ Journal editors can address these gaps by holding authors accountable for adhering to CONSORT guidelines on reporting of participant flow, a goal that may require an intervention as simple as an email reminder to peer reviewers.^157^

### Heterogeneity in Reporting of Reasons for Noninclusion of Eligible Infants

We found wide variability in how studies reported the reasons that eligible infants were not included. Included studies described reasons for noninclusion with varying level of detail, used nonspecific language to describe reasons, or combined disparate reasons into a single category. Consequently, classifying reasons often required subjective interpretation. For example, 6 articles^31,89,96,100,112,121^ in our review referred to “parents not approached or declined” as a single reason for noninclusion, even though these are fundamentally different reasons that call for unique changes in practice to optimize inclusion. Furthermore, some studies gave vague reasons for noninclusion of infants, such as “nonqualifying exclusion” or “other nonmedical reasons,” which were difficult to interpret. Our findings are similar to a review of adult palliative care trials,^158^ which found that more than one-half of reasons for noninclusion of participant data were unclassified.

Clear, transparent, and complete reporting of which patients are and are not included in trial results is essential for assessing the validity and generalizability of findings^154,159,160^ and for promoting health equity.^161^ Previous literature suggests that participants in both adult^10,159^ and neonatal^6,8,9^ trials tend to be healthier and more socioeconomically privileged compared with nonparticipants. The exclusion of sicker participants and those with greater socioeconomic risk factors can lead trialists to overestimate the effect size of interventions.^162^ Beyond this, systematic underrepresentation of racially and ethnically marginalized populations in neonatal research threatens to exacerbate existing problematic health care disparities.^163,164,165^ Equity concerns occur throughout the process of trial recruitment in pediatric research^166^ and are inextricable from questions about study generalizability.^161^

CONSORT provides valuable guidance on reporting for clinical trials, but we agree with others^167^ who call for more detailed guidance on how to report reasons for noninclusion of eligible patients. Standardized categories of reasons for noninclusion may ease the burden for authors trying to clearly report participant flow and help clinicians interpret and compare trial findings. We propose that categories similar to the 10 that we used in our review could be incorporated into the next iteration of CONSORT guidelines with clear definitions for each category.

### A Need for Ongoing Work to Understand Why Parents Decline or Are Not Approached for Consent

We found that modifiable or potentially modifiable factors accounted for almost 94% of the reasons eligible infants were not included in reported trial results. Of these, parents declining was by far the most frequently cited reason, followed by parents not being approached.

Recent studies have investigated factors influencing parents’ decisions about whether or not to participate in neonatal trials.^168,169^ A sense of altruism and potential for benefit to the infant may motivate families to participate.^170^ Trust or distrust in the medical system and relationship building between researchers, clinicians, and families are also important factors.^169,170,171,172,173^ Our findings highlight the importance of continuing work to understand why parents decline to participate in neonatal trials. Including parents who chose not to participate in a clinical trial can be very challenging; they are more likely to decline studies about their recruitment experience and researchers or regulators may be hesitant to approach them.^168^ Nonetheless, including the views of neonatal research decliners is critically important, and studies that have prioritized this population have succeeded in including them.^9,169,170,171^

Medical researchers and funders across many domains have identified improving participation in clinical trials as a high priority need.^174,175^ Many of these efforts, such as very large All of Us Research Program,^176^ strive to decrease disparities in who participates in research, with a stated goal to increase participation of groups historically under-represented in biomedical research. Within neonatal research, members of our team have developed and are testing a researcher facing intervention with similar goals.^177^ To be done ethically, such efforts must have strong ethical oversight, including in content of intervention, in stakeholder engagement, and in goal setting. While there is strong and clear ethical justification for researchers in neonatology and elsewhere to improve diversity of the research population, this cannot be the only goal or measured outcome. Such work must aim to improve the parental experience of recruitment and support an enrollment decision better aligned with parental values.

In addition, our findings highlight the importance of further investigating why some families are never approached to participate in neonatal trials. Although few explicit explanations for why a family was not approached were given in the articles included in this review, we can imagine several plausible reasons. For example, research staff may be less likely to approach families if there are language differences, if there are challenges contacting the family, if there is a short or inconvenient enrollment window (eg, overnight), or if the clinical team is not aligned with the research team’s goals. Reasonable accommodations can be made to mitigate some of these challenges, such as hiring interpreters or multilingual research staff. Several researchers and bioethicists are studying how to contend with short enrollment windows for conditions requiring emergency treatment, for example, through the use of deferred consent for investigational treatments of hypoxic-ischemic encephalopathy.^178,179,180,181,182^ Nonetheless, the first step is to better understand why some families are never approached, and future work should be directed toward this goal.

### Neonatal Clinical Trial Results and Eligible Participants’ Recruitment

Among the articles included in this review, we found that less than one-half of eligible infants were represented in trial results. The proportions of eligible patients included in large pediatric intensive care unit trials are similar.^183,184,185,186^ This is particularly problematic because many neonatal trials have been underpowered or terminated early due to recruitment failures.^11,12,13,14,15,16,17^ Trialists and funders can use the data presented in this review to inform realistic expectations about participant accrual and define inclusion and exclusion criteria, frame enrollment windows, and hire staff accordingly.^149^ One mechanism trialists may consider for increasing enrollment is coenrollment in multiple trials because this seems to be accepted by families.^181,187^ It is interesting to note that, in our review, trials of surgical interventions, those without dedicated funding, those that took place at 5 or fewer sites, or those that enrolled for more than 28 months had higher proportions of eligible infants included in reported results. Future work may aim to corroborate these findings and better understand why these trends emerged.

### Limitations

This review is limited by how the included studies reported participant flow. The heterogeneity in how studies classified reasons for noninclusion made it challenging to extract and compile data reproducibly. To reduce bias, our team extracted data and categorized reasons for noninclusion based on consensus among at least 3 members; however, we acknowledge that a degree of subjectivity is inevitable. Future work using larger numbers of clinical trials must evaluate how the components of the research, such as interval risk over baseline, burdens of participation to families, duration of enrollment window, or number of sites participating, may impact reasons for noninclusion. The generalizability of our finding outside the US context may be limited. Future work should assess reasons for noninclusion internationally as well as potential differences between countries.

## Conclusions

This systematic review of participant flow through neonatal clinical trials found that, in aggregate, fewer than one-half of eligible participants were included in reported results and that interpreting the reasons why they were not recruited as participants was challenging due to heterogeneity in reporting. Based on these findings, we recommend that future iterations of CONSORT guidelines offer standard categories for reporting reasons for noninclusion. In addition, we note that, despite heterogeneity in reporting, it was clear that the majority of reasons for noninclusion were modifiable or potentially modifiable, particularly because parents declined or were not approached; future work aimed at understanding how to increase trial participation should focus on these reasons. Finally, our findings illustrate that participant accrual in neonatal trials is a slow process, which trialists should account for as they design future studies.

## Notes

Supplement 1. **eTable.** Summary of Studies

Supplement 2.
Data Sharing Statement
