# Supplementary material for: Eligible Infants Included in Neonatal Clinical Trials and Reasons for Noninclusion: A Systematic Review
Source: JAMA Netw Open. 2024 Oct 25;7(10):e2441372. doi: 10.1001/jamanetworkopen.2024.41372 (PMC11581680; doi:10.1001/jamanetworkopen.2024.41372)
Supplement: Supplement 1. — eTable. Summary of Studies [file jamanetwopen-e2441372-s001.pdf]

Supplemental Online Content

Shaikh H, Lyle ANJ, Oslin E, Gray MM, Weiss EM. Eligible infants included in neonatal clinical trials and resons for noninclusion: a systematic review. *JAMA Netw. Open*. 2024;7(10):e2441372. doi:10.1001/jamanetworkopen.2024.41372

eTable. Summary of Studies

This supplemental material has been provided by the authors to give readers additional information about their work.

© 2024 Shaikh H et al. *JAMA Network Open.*

eTable. Summary of Studies

First Author, Year	Study Summary	Study Type	Prenatal Enrollment (Y/N)	Sites (N)	Enrollment Duration (months)	Funding Source	Flow Diagram Included (Y/N)	Patients Screened (N)	Patients Eligible (N)	Patients Allocated to Intervention (N)	Patients Included in Primary Outcome (N)	Reasons for Non-inclusion	Proportion of Eligible Patients Included in Results	Participant Accrual Rate
Abbey, 2021	RCT evaluating use of ECG for heart rate assessment of preterm neonates during delivery room resuscitation to no ECG use for impact on time to heart rate ≥100bpm	DR	N	1	10	Gov	Y	NR	71	51	51	13 Study factor (13 "research equipment failure")<br>7 Not approached (7 "missed enrollment”)	72%	5.1
Alrifai, 2018	RCT comparing the use of a parenteral protein calculator clinical decision support system to standard care for attaining appropriate total protein intake in preterm infants	Gen	N	1	3	Gov, Hosp	N	NR	NR	42	42	N/A		14.0

© 2024 Shaikh H et al. *JAMA Network Open.*

Amaro, 2018[1]	RCT evaluating the effect of early caffeine compared to placebo administration on age of first successful extubation in preterm infants	Gen	N	1	36	NR	Y	208	143	87	83	1 Study factor ("extubation and administration of open-label caffeine by the clinical team before starting the administration of the assigned study drug.")<br>31 Parents declined<br>24 Parents not approached ("unable to request consent")<br>1 Clinician refused<br>2 Consent withdrawn (by parent)<br>1 Death	58%	2.3
Amatya, 2017	RCT to determine whether gradual or sudden weaning from CPAP to room air is more successful	Gen	U	1	26	NR	Y	154	132	70	68	2 Study factor (2 "protocol deviation")<br>12 Parents declined<br>40 Parents not approached (32 "unable to consent," 2 "not approached")<br>10 Clinical reason	52%	2.6
Anderson-Berry, 2017	RCT to determine the effects of two different vitamin D3 dosage schemes on serum vitamin D concentration, growth, PTH normalization, and bone density among preterm infants	Gen	U	1	NR	Gov, Hosp	Y	369	54	32	32	21 Parents declined<br>1 Other	59%	

© 2024 Shaikh H et al. *JAMA Network Open.*

Angeles, 2020	Prospective, randomized trial comparing 30% oral dextrose, facilitated tucking, and 30% oral dextrose plus facilitated tucking for pain measured through premature infant pain profile-revised (PIPP-R)	Gen	N	1	51	Gov	Y	193	169	169	152	3 Consent withdrawn (by parent)<br>14 Clinical reason (6 "acuity changed," 8 "line did not draw")	90%	3.0
Angeles, 2021	RCT comparing urinary markers of ATP metabolism (hypoxanthine, xanthine, uric acid) in preterm neonates who received oral sucrose, oral dextrose, or standard of care prior to painful procedures	Gen	N	1	32	Gov	N	NR	NR	51	46	4 Study factor (3 study drug not administered with appropriate timing, 1 study drug inappropriately administered to a patient in the control group)<br>1 Clinical reason		1.4
Arnold, 2020	A dose-finding randomized trial of cycled compared to continuous phototherapy for mean peak total serum bilirubin level, total phototherapy hours within 14 days, and predischarge wave V latency BAERs	Gen	U	6	56	Gov	Y	727	612	305	256	34 Study factor (34 decision during the study to drop one arm of included patients because an interim analysis showed no difference in effect)<br>160 Parents not approached (101 "consent not requested" because of study funding and personnel limitations, 50 "parent unavailable," 9 "not approached")<br>147 Parents declined<br>15 Death	42%	0.8

© 2024 Shaikh H et al. *JAMA Network Open.*

Atchley, 2019	RCT to investigate the effect of an enhanced compared to standard protein diet on weight gain and fat accretion over the 4-week study period among preterm infants <32 weeks who had attained full enteral feeds	Gen	N	1	20	NR	Y	345	173	36	33	75 Parents not approached (75 "no family contact")<br>24 Parents declined<br>6 Participating in another study<br>2 Consent withdrawn (by parent)<br>7 Clinical reason (6 transferred to another facility, 1 clinical change)<br>17 Death<br>9 Other	19%	1.7
Backes, 2020	Randomized trial comparing rates of CPAP failure between two CPAP modes (Seattle-PAP versus Fisher & Paykel-CPAP) among infants born <30 weeks	Gen	N	5	22	Priv	Y	NR	309	232	232	46 Parents declined<br>25 Participating in another study<br>6 Clinical reason (6 patients who were consented but remained intubated and therefore could not be placed on CPAP)	75%	2.1
Balakrishnan, 2018	RCT comparing the effects of lower- and higher-dose parenteral amino acids on growth and neurodevelopmental outcomes among very low birth weight infants	Gen	N	1	49	NR	N	467	425	168	116	90 Study factor (90 "research staff unavailable")<br>65 Parents not approached (65 "parent unavailable")<br>102 Parents declined<br>19 Death<br>33 Other (lost to follow-up)	27%	2.4

© 2024 Shaikh H et al. *JAMA Network Open.*

Ballengee, 2018	RCT comparing the effects of erythromycin versus placebo on esophageal reflux in preterm neonates.	Gen	N	1	24	Ind, priv	Y	NR	NR	43	31	3 Study factor (3 "malpositioned probes")<br>8 Clinical reason (7 patients who were consented for measurement of gastroesophageal reflux events but did not meet severity criteria for inclusion in analysis, 1 patient who developed sepsis)<br>1 Death		1.3
Bedwell, 2021	RCT evaluating warming human milk continuously versus with a hot water bath warming before feeding among preterm infants for effect on weight gain	Gen	U	1	NR	NR	Y	47	46	45	44	1 Parent declined<br>1 Clinical reason (developed necrotizing enterocolitis)	96%	
Blakely, 2021	RCT evaluating initial laparotomy versus peritoneal drainage for impact on death or neurodevelopmental impairment in preterm infants with necrotizing enterocolitis or isolated intestinal perforation	Surg	N	20	87	Gov	Y	1398	992	310	295	13 Study factor (13 incomplete or absent neurodevelopmental assessment at follow-up)<br>149 Parents not approached (66 "parent unavailable," 83 "consent not requested")<br>245 Parents declined<br>232 Clinician declined<br>2 Consent withdrawn (unspecified by whom)<br>56 Other (52 "other," 4 "consented but not randomized")	30%	0.2

© 2024 Shaikh H et al. *JAMA Network Open.*

Bogen, 2018	Feasibility trial evaluating the impact of 24 compared to 20 kilocalorie/ounce formula on weight trajectory of infants prenatally exposed to methadone	Gen	Y	1	25	Gov	Y	NR	NR	85	49	2 Parents declined (1 "incomplete consent," 1 "mother left infant with no further contact")<br>2 Consent withdrawn (by parent)<br>8 Clinical reason (8 patients who were consented prenatally but did not deliver at the study hospital)<br>24 Consented prenatally and no longer eligible at time of allocation to intervention		2.0
Brandon, 2017	RCT to determine the effect of early (28 weeks PMA) versus late (36 weeks PMA) initiation of cycled light on weight gain during hospitalization and length of stay	Gen	N	1	41	Gov	Y	193	193	121	118	35 Parents declined34 Participating in another study2 Clinical reason (2 transferred to another facility)1 Death3 Other (3 unaccounted for)	61%	2.9
Brion, 2020	Randomized trial evaluating the impact of advancing human milk fortification based on serial milk nutrient analyses compared to standard advancements on weight gain and linear growth from birth to 36 weeks postmenstrual age or discharge	Gen	N	1	31	Ind, Hosp	Y	293	230	120	116	110 Parents declined<br>4 Deaths	50%	3.7

© 2024 Shaikh H et al. *JAMA Network Open.*

Brusseau, 2020	Randomized trial comparing total morphine treatment days among infants with neonatal abstinence syndrome receiving clonidine versus phenobarbital as adjunctive therapy due to inadequate treatment with morphine alone	Gen	N	1	6	NR	Y	78	47	26	25	2 Study factor (2 inappropriately received intravenous morphine)<br>8 Parents not approached (8 "unavailability of parent or study staff")<br>11 Parents declined<br>1 Consent withdrawn (by parent)	53%	4.2
Bruzoni, 2017[1]	RCT to evaluate the impact of sutureless versus sutured gastroschisis closure on time to extubation and time to full feeds.	Surg	Y	1	66	NR	Y	62	56	39	39	17 Parents declined	70%	0.6
Butler-O'Hara, 2017	RCT to determine whether suppository use among infants with hyperbilirubinemia shortens phototherapy duration compared to no suppository use	Gen	N	1	15	NR	Y	391	275	79	79	162 Not approached (162 "inability to obtain consent before the neonate started phototherapy")<br>34 Parents declined	29%	5.3
Calkins, 2017	RCT to evaluate the effects of low- versus high-dose soybean-based intravenous lipid emulsions on development of cholestasis among neonates with gastrointestinal disorders.	Gen	N	2	41	Gov	Y	87	87	41	36	33 Not approached (33 combined reason of "parent/ legal guardian not available, missed by investigator because of weekend/ holiday")<br>13 Parents declined<br>5 Other (5 unaccounted for)	41%	0.4

© 2024 Shaikh H et al. *JAMA Network Open.*

Ceyhan-Birsoy, 2019	RCT to evaluate the impact of newborn genomic sequencing compared to standard care (state-mandated newborn screening and family history-based genetic counseling) on clinical care, parent and clinician behaviors, and economic outcomes in all healthy and ill newborns (only NICU infants included in our review)	Gene	N	2	22	Gov	Y	NR	436	45	32	152 Parents declined/not approached (152 "non-responsive or unable to coordinate consent before discharge")<br>239 Parents declined<br>13 Other (13 unaccounted for)	7%	0.7
Choi, 2019	Prospective study evaluating the impact of T2 magnetic resonance in predicting postsurgical bleeding for term infants undergoing cardiopulmonary bypass for repair of congenital heart disease	Surg	U	1	17	Ind	N	NR	NR	100	92	8 Study factor (8 "missing data")		5.4
Cholette, 2017	RCT to assess safety, daily hemoglobin concentration, and oxygen utilization among infants with congenital heart disease requiring surgical management managed with a liberal versus conservative red blood cell transfusion strategy	Surg	N	1	29	Gov	Y	NR	211	165	162	5 Parents not approached (5 "no family available")<br>41 Parents declined<br>3 Clinical change (3 needing to transition from cardiopulmonary bypass to extracorporeal membrane oxygenation)	77%	5.6

© 2024 Shaikh H et al. *JAMA Network Open.*

Chorna, 2018	Quasi-randomized prospective study comparing active, suck-contingent exposure to mother's voice to standard care on speech-sound differentiation in preterm infants	Gen	N	1	NR	Gov	N	NR	NR	20	20	N/A		
Claassen, 2019[1]	RCT evaluating the impact of extubation to one of two different bubble CPAP devices (B&B Bubbler©, BabiPlus©) on extubation failure in newborns with birthweight 500-1250g who received surfactant within the first 24 hours of life	Gen	N	1	16	Hosp	N	NR	NR	42	42	N/A		2.6
Cook, 2017	RCT to assess the impact of sucrose solution compared to placebo on infant pain during peripheral IV insertion	Gen	N	1	NR	NR	Y	NR	93	86	40	46 Clinical reason (40 peripheral IV not needed, 6 multiple peripheral IV attempts)<br>7 Other (7 combined reason provided of "did not meet eligibility criteria or declined")	43%	
Cooper, 2020	RCT comparing 15-20 minutes of daily developmentally appropriate motor activities to standard care among preterm infants for effect on body composition at one year of life	Gen	N	3	NR	Gov	Y	233	216	163	99	10 Study factor (10 patients who did not receive the allocated intervention)<br>42 Parents declined<br>28 Consent withdrawn (by parent)<br>39 Other (11 "other," 28 loss to follow-up)	46%	

© 2024 Shaikh H et al. *JAMA Network Open.*

Corrigan, 2020	Randomized trial comparing exposure of preterm infants to music therapy (recorded maternal singing or heartbeat) to standard care for effect on pain measured via premature infant pain profile (PIPP) during and after retinopathy of prematurity exam	Gen	N	1	NR	Hosp	Y	NR	NR	100	74	23 Study factor (23 pain scale presumably not performed)<br>3 Consent withdrawn (by clinician)		
Cummings, 2020	RCT comparing aerosolized calfactant administration to usual care among infants with respiratory distress syndrome for effect on frequency of clinically required intubation and surfactant instillation within the first 4 days of life	Gen	Y	22	15	Ind	Y	NR	772	457	457	20 Study factor (20 patients consented as part of a different cohort that was not included in the results)<br>108 Parents declined<br>187 Other (187 patients not included with the following reason given, which we found difficult to interpret: "presumed that most never met entry criteria; however, some consented patients may have not been randomly assigned for other reasons.")	59%	1.4

© 2024 Shaikh H et al. *JAMA Network Open.*

Czarnecki, 2020[1]	RCT comparing the use of parent/nurse controlled analgesia to continuous opioid infusion on the outcome of average opioid consumptions up to 3 post-operative days	Surg	Y	1	25	NR	Y	371	108	36	25	42 Study factor (31 "logistics," 11 "did not receive allocated intervention")<br>35 Parents declined (7 "refusal to hear about study," 28 "declined")<br>6 Clinical reason (6 patients who were approached for consent prior to surgery but no longer met inclusion criteria after the surgery)	23%	1.0
Czynski, 2020	Randomized trial comparing treatment of neonatal abstinence syndrome with morphine versus methadone for impact on neurodevelopmental outcomes, measured as NICU Network Neurobehavioral Scale at hospital discharge and Bayley Scale and Child Behavior Checklist at 18 months	Gen	U	8	37	NR	Y	NR	NR	183	99	17 Study factor (17 "infant not in correct state at time of exam")<br>1 Consent withdrawn (by parent)<br>66 Consented prenatally and no longer eligible at time of allocation to intervention		0.3
Daniel, 2020	RCT comparing aromatherapy plus standard care versus standard care alone for effect on duration of therapy and length of stay among neonates with opioid withdrawal syndrome	Gen	N	1	21	NR	N	NR	NR	38	38	N/A		1.8

© 2024 Shaikh H et al. *JAMA Network Open.*

Davidson, 2019	RCT to evaluate the impact of olfactory stimulation with mother's milk compared to water (sham) on post-menstrual age at attainment of full oral feeds among preterm infants born 28 0/7 - 33 6/7 weeks	Gen	N	1	NR	NR	N	NR	NR	36	33	1 Consent withdrawn (by parent)<br>2 Clinical reason		
Davis, 2018	RCT comparing safety and efficacy of methadone to morphine for treatment of neonatal abstinence syndrome	Gen	U	8	37	Gov	Y	NR	NR	183	116	1 Consent withdrawn (by parent)<br>66 Consented prenatally and no longer eligible at time of allocation to intervention		0.4
Detmer, 2020	RCT comparing music therapy (multimodal neurological enhancement) to standard care for effect on post-discharge Mullen Scales of Early Learning scores among preterm neonates	Gen	N	1	22	None	N	NR	NR	84	48	1 Consent withdrawn (by parent)<br>9 Clinical reason (9 "transfer to another facility or medical instability")<br>26 Other (26 loss to follow-up)		2.2
DuPont, 2021	RCT assessing the safety and feasibility of administering 1 dose of darbepoetin-α vs placebo to infants ≥34 weeks gestation with mild neonatal encephalopathy based on occurrence of adverse events and Bayley Scale score at 8-12 months corrected age	Gen	N	4	18	Gov	Y	78	29	21	20	8 Parents declined<br>1 Other (a patient that was not clearly accounted for but was possibly lost to follow-up)	69%	0.3

© 2024 Shaikh H et al. *JAMA Network Open.*

Elzein, 2020	RCT comparing nitric oxide versus placebo added to oxygenator during cardiopulmonary bypass during Norwood procedure for effect on serum markers of ischemia/reperfusion injury	Surg	N	1	51	None	N	NR	NR	25	24	1 Clinical reason (1 diagnosis with a genetic syndrome)		0.5
Emery, 2019	RCT evaluating the differential impact of 2 music therapy regimens (intensive-intermittent vs/ standard spaced) on developmental milestone acquisition over the 4 week study period in infants >44 weeks PMA	Gen	N	1	NR	Gov	N	NR	NR	27	24	3 Clinical reason		
Eze, 2018	RCT comparing a sprinting versus non-sprinting protocol for successful CPAP weaning to room air	Gen	N	2	NR	NR	Y	152	97	86	80	11 Parents declined<br>5 Consent withdrawn (3 by parent and 2 by clinician)<br>1 Clinical reason (1 patient who was extubated directly to nasal canula)	82%	
Foglia, 2019	Safety and feasibility trial of intubation prior to umbilical cord clamping at delivery in infants >= 36 weeks with congenital diaphragmatic hernia	DR	Y	1	9	Gov, Ind	Y	30	26	20	20	5 Parents declined (4 "declined," 1 "no decision")<br>1 Consented prenatally and no longer eligible at time of allocation to intervention	77%	2.2

© 2024 Shaikh H et al. *JAMA Network Open.*

Forde, 2020	RCT comparing urinary biomarkers of energy utilization and oxidative stress among preterm infants who received 1 hour of kangaroo mother care versus incubator care on day 3 of life	Gen	N	1	6	Gov, Priv	Y	NR	61	51	51	5 Parents declined<br>5 Consent withdrawn (reason given: "withdrew because they became ill or changed their mind")	84%	8.5
Frost, 2021	RCT to assess feasibility of providing concentrated emulsified long-chain polyunsaturated fatty acids (LCPUFA) in two different doses compared to placebo and to assess the impact on blood LCPUFA concentrations among preterm infants	Gen	N	2	13	Ind	Y	80	67	30	30	28 Parents declined<br>9 Other	45%	1.2
Gautam, 2020	RCT comparing the effect of platelet transfusion during the rewarming phase of cardiopulmonary bypass to transfusion at the end of bypass on need for postoperative blood product resuscitation	Surg	N	1	19	None	N	NR	NR	46	42	4 Clinical reason (4 "postoperative need for extracorporeal support")		2.2

© 2024 Shaikh H et al. *JAMA Network Open.*

Gaynor, 2018	RCT evaluating the effect of remote ischemic preconditioning compared to sham procedure on postoperative white matter injury among neonates undergoing cardiopulmonary bypass	Surg	N	1	58	Gov, Priv	N	112	NR	67	50	4 Study factor (4 "technical issues with the MRI scanner")<br>5 Consent withdrawn (by parent)<br>8 Clinical reason (1 patient with an MRI finding that delayed surgery, 7 patients with failure to complete the second MRI due to clinical status)		0.9
Gerges, 2018	RCT evaluating the impact of initiation of oral feeds at 30 compared to 33 weeks postmenstrual age on time to full oral feedings or hospital discharge	Gen	U	1	32	Hosp	Y	378	84	66	65	4 Parents not approached (4 "not able to be approached")<br>14 Parents declined<br>1 Clinical change (transferred to another facility)	77%	2.0
Glass, 2017	RCT to evaluate the impact of oropharyngeal administration of colostrum versus sterile water on salivary IgA levels, incidence of late-onset sepsis, and necrotizing enterocolitis	Gen	N	1	NR	Gov	Y	93	50	30	30	8 Parents not approached (8 "not approached")<br>12 Parents declined	60%	

© 2024 Shaikh H et al. *JAMA Network Open.*

Graham, 2019	RCT to evaluate the impact of intraoperative methylprednisone compared to placebo on postoperative morbidity and mortality among term infants undergoing cardiopulmonary bypass for repair of congenital heart disease	Surg	U	2	65	Gov	Y	NR	233	190	176	1 Consent withdrawn (by parent)<br>12 Clinical reason<br>1 Consented prenatally and no longer eligible at time of allocation to intervention (the reason given was "prematurity;" assumed that this patient was therefore consented prenatally and no longer eligible after birth)<br>43 Other (43 unaccounted for)	76%	1.4
Gray, 2017[1]	RCT comparing every three-hour to every six-hour oral feeding attempts to determine which schedule allows for more rapid attainment of full oral feeding among neonates born preterm	Gen	N	2	9	NR	Y	161	87	55	52	12 Study factor (12 patients who were started on oral feeds before being approached about the study)<br>20 Parents declined<br>1 Withdrawn (by parent)<br>3 Clinical reason (3 patients who did reach full feeds due to chronic aspiration)	60%	2.9
Gupta, 2021	Blinded, randomized trial comparing administration of soy-based intravenous lipid emulsion in two concentrations to infants with gastrointestinal surgical disorders for impact on rate of rise of direct bilirubin and incidence of intestinal failure-associated fatty liver disease	Gen	N	1	81	Hosp	Y	73	63	50	40	12 Parents declined<br>11 Clinical reason	63%	0.5

© 2024 Shaikh H et al. *JAMA Network Open.*

Hammer, 2019	RCT to evaluate the efficacy of intravenous acetaminophen at two different dosing regimens compared to placebo in controlling pain among neonates >= 28 weeks and infants postoperatively or following trauma	Gen	N	22	36	Ind	N	NR	NR	215	197	18 Study factor (18 patients who are presumed to not have "received a dose of study drug and completed at least 1 assessment"		0.2
Hammond, 2021	RCT evaluating the amino acid composition of total parenteral nutrition (18% vs. 12.5% amino acids (experimental vs standard, respectively)) among VLBW infants for impact on sleep state distribution after achieving full feeds	Gen	N	1	NR	Gov	N	NR	178	115	71	63 Parents declined<br>32 Clinical reason (1 transferred to another facility, 31 clinical change)<br>12 Death	40%	
Hart, 2017	RCT evaluating the impact of Velcro versus twill tie for securing tracheostomy tubes on skin-related complications and accidental decannulation	Surg	N	1	19	Gov	Y	93	78	63	57	12 Parents declined<br>6 Clinical reason (5 patients who did not undergo tracheotomy, 1 patient diagnosed with a genetic skin condition)<br>3 Other	73%	3.0

© 2024 Shaikh H et al. *JAMA Network Open.*

Havranek, 2019	RCT to evaluate the impact of prone compared to supine positioning on need for respiratory intervention following scheduled cesarean delivery of vigorous newborns at 37-42 weeks	DR	Y	1	32	NR	Y	NR	658	500	456	48 Study factor (38 "research team absence," 3 "protocol violation," 7 "research staff not called")<br>99 Parents declined<br>11 Participating in another study<br>10 Shortened consent window (10 "emergent delivery")<br>2 Clinical reason (2 "congenital defects known after delivery")<br>32 Consented prenatally and no longer eligible at time of allocation to intervention	69%	14.3
Hibbs, 2018	RCT evaluating the effectiveness of two different vitamin D dosing strategies in preventing recurrent wheezing among Black preterm infants after hospital discharge	Gen	N	4	37	Gov	Y	1481	1161	300	269	440 Parents not approached (440 "not approached")<br>421 Parents declined<br>18 Consent withdrawn (by parent)<br>1 Death<br>12 Other (12 loss to follow-up)	23%	1.8

© 2024 Shaikh H et al. *JAMA Network Open.*

Jadcherla, 2020[1]	RCT comparing four weeks of a feeding bundle (total fluid limitation, feeds over 30 minutes in right lateral position, supine postprandial position) plus acid suppression versus acid suppression alone for impact on GERD symptom scores and independent oral feeding skills among infants 34-60 weeks post-menstrual age	Gen	N	1	75	Gov	Y	688	178	76	76	55 Parents declined<br>47 Other	43%	1.0
Jakubowitz, 2018	Trial comparing the accuracy of transcutaneous CO_2 and O_2 monitoring electrodes set to various temperatures	Gen	N	1	NR	Hops	N	NR	NR	20	20	N/A		
Jooste, 2019	RCT to evaluate the impact of intraoperative antithrombin administration compared to placebo on postoperative coagulability status among infants birth to 7 months undergoing cardiopulmonary bypass for repair of congenital heart disease	Surg	U	2	24	Ind	N	223	68	40	39	1 Clinical reason<br>28 Other (28 unaccounted for)	57%	0.8

© 2024 Shaikh H et al. *JAMA Network Open.*

Josephsen, 2020[1]	RCT comparing umbilical cord milking versus immediate cord clamping among extremely preterm infants for effect on initial hemoglobin level and need for blood transfusions in the first 28 days of life	DR	Y	1	NR	NR	Y	203	144	59	56	1 Study factor (1 "OB unaware subject was in study)<br>11 Parents declined<br>8 Shortened consent window (8 "delivered too quickly)<br>1 Clinical change (1 transferred to another facility)<br>62 Consented prenatally and no longer eligible at time of allocation to intervention<br>1 Death<br>4 Other	39%	
Juul, 2020	RCT evaluating administration of intravenous high-dose erythropoietin to placebo in extremely preterm infants for impact on composite outcome of death or severe neurodevelopmental impairment at 22 to 26 months of age	Gen	Y	19	34	Gov	Y	3266	2233	941	741	170 Study factor (89 "did not have research team available," 2 "had previous administration of erythropoietin," 1 "complication with consent," 25 "incomplete examination," 53 "did not have examination within window")<br>1016 Parents declined<br>55 Participating in another study<br>5 Patient death<br>246 Other (85 "not expected to be available for follow-up," 44 "other," 117 loss to follow-up)	33%	1.1

© 2024 Shaikh H et al. *JAMA Network Open.*

Kakkilaya, 2021	Randomized trial comparing immediate versus stepwise discontinuation of CPAP on total CPAP days among preterm infants	Gen	N	1	42	Hosp	Y	488	350	226	226	59 Not approached (59 "missed")<br>65 Parents declined	65%	5.4
Katheria, 2017	Evaluation of a subset of infants included in Neu-Prem Trial RCT to evaluate whether displaying compared to not displaying EKG information during delivery room resuscitation impacted time to first change in FiO2, first positive pressure ventilation, first increase in airway pressure, and first intubation	DR	Y	1	NR	NR	Y	200	197	40	40	98 Study factor (8 "equipment not available, "41 "research staff not available," 1 "equipment failure," 40 "equipment not available/not placed," 6 "EKG signal inadequate," 2 "corrupt data")<br>10 Parents declined<br>1 Death<br>43 Other (2 "preconsented but unable to enroll," 41 unaccounted for)<br>5 Shortened consent window (5 "precipitous delivery")	20%	

© 2024 Shaikh H et al. *JAMA Network Open.*

Katheria, 2021	RCT comparing ECG integrated into Panda Bed Warmer versus stand-alone cardiac monitor for time to heart display following electrode placement during resuscitation of preterm infants	DR	Y	1	5	Ind	Y	147	143	42	42	11 Study factor (2 "research staff not available," 6 "discharged before follow-up," 2 "research staff unavailable at time of delivery," 1 "research bed not available at the time of delivery")<br>5 Parents not approached (5 "no consent prior to delivery")<br>11 Parents declined (11 patients with combined category of "parents not approached/declined")<br>2 Shortened consent window (2 emergency cesarean section)<br>50 Consented prenatally and no longer eligible at time of allocation to intervention<br>22 Other (16 "other," 6 "study completed enrollment before delivery")	29%	8.4

© 2024 Shaikh H et al. *JAMA Network Open.*

Kim, 2020	RCT evaluating the effect of withholding the initial 48 hours of empiric antibiotics in low-risk preterm infants on microbiome diversity measured through DNA sequencing of fecal sampling	Gen	U	1	NR	Gov	Y	NR	40	27	22	7 Study factor (2 patients for whom an initial CBC did not result in time for recruitment, 5 patients who "did not receive assigned intervention (pharmacy error)")<br>11 Consented prenatally and no longer eligible at time of allocation to intervention	55%	
Kingsmore, 2019	RCT to evaluate the diagnostic effectiveness and clinical utility of two rapid genomic sequencing methods (rapid whole genome sequencing, rapid whole exome sequencing) among infants <4 months old admitted to the NICU, PICU, or CVICU with features suggestive of a genetic illness	Gene	N	1	15	Gov, Priv, Hosp	Y	1248	685	213	213	216 Not approached (31 "unable to obtain permission," 81 ">96 hours elapsed since admission," 87 "discharged," 17 "unable to meet family")<br>178 Parents declined<br>61 Clinician refused<br>4 Death<br>13 Other	31%	14.2
Kirpalani, 2020	RCT comparing higher or lower threshold for red-cell transfusion (based on corrected gestational age and respiratory support) among extremely low birth weight neonates for impact on composite outcome of death or neurodevelopmental impairment at 22 to 26 months of age	Gen	N	19	63	Gov	Y	4915	3958	1824	1692	326 Parents not approached (326 “not approached”)<br>1404 Parents declined<br>27 Clinician refused<br>10 Consent withdrawn (unclear whether by parent or clinician)<br>57 Death<br>442 Other (320 “other,” 33 “incomplete follow-up,” 79 “assessment of vital status only,” 10 loss to follow up)	43%	1.4

© 2024 Shaikh H et al. *JAMA Network Open.*

Kochan, 2019	RCT to evaluate the impact of elevated compared to flat head positioning on incidence of intraventricular hemorrhage among ELBW (<1000g) neonates	Gen	N	1	38	NR	Y	354	303	180	180	113 Study factor (113 "investigator unavailable")<br>10 Parents declined	59%	4.7
Kotloff, 2019	RCT to evaluate the impact of mupirocin compared to no treatment on *Staphylococcus aureus* decolonization among NICU infants <24 months old	Gen	N	6	25	Gov	Y	6327	442	155	130	5 Study factor (4 "no pretreatment culture," 1 "baseline culture out of window")<br>199 Parents declined/not approached (199 "parent refused or not located")<br>69 Clinician refused<br>12 Clinical reason (12 "negative results on pretreatment cultures")<br>27 Other (19 "other," 8 combined reason of study factor or clinical change: "no day 8 culture or clinical SA infection before day 8")	29%	0.9
Kraft, 2017[1]	RCT comparing the effect of sublingual buprenorphine versus oral morphine on duration of treatment for neonatal opioid withdrawal syndrome	Gen	U	1	56	Gov	Y	121	63	63	58	5 Consent withdrawn (by parent)	92%	1.0

© 2024 Shaikh H et al. *JAMA Network Open.*

Kumar, 2017	Prospective trial comparing breast milk fortification with Enfamil human milk fortifier acidified liquid versus Similac human milk fortifier hydrolyzed protein concentrated liquid on weight gain and metabolic acidosis	Gen	U	1	NR	NR	N	NR	NR	31	31	N/A		
Lafferty, 2021	RCT evaluating exposure to Mozart for two weeks compared to no exposure on outcome of time to regain birthweight among preterm infants	Gen	N	1	15	None	Y	70	59	40	40	7 Parents not approached (7 ">3 days of life" (timing out of eligibility period before study team could approach))<br>7 Parents declined<br>1 Participating in another study<br>4 Other (1 "concerns about mother's ability to consent," 3 "unable to follow-up")	68%	2.7
Lam, 2020	RCT evaluating CPAP extended for 2 additional weeks to standard discontinuation of CPAP for impact on functional residual capacity at the end of the 2-week treatment period and at hospital discharge among neonates born ≤32 weeks requiring CPAP ≥24 hours	Gen	N	1	21	Gov	Y	116	75	50	44	4 Study factor (4 "unacceptable PFT")<br>25 Parents declined<br>1 Consent withdrawn (unspecified by whom)<br>1 Clinical reason (1 patient who failed discontinuation of CPAP)	59%	2.1

© 2024 Shaikh H et al. *JAMA Network Open.*

Lambert, 2017	Safety and feasibility trial for a passive range of motion exercise program for infants with congenital heart disease	Surg/ Gen	N	3	13	Gov	Y	59	25	20	19	5 Parents declined<br>1 Consent withdrawn (by parent)	76%	0.5
Laptook, 2017	RCT to evaluate the effect of therapeutic hypothermia initiated 6 to 24 hours after birth compared to standard care on death and neurodevelopmental outcomes among infants with hypoxic ischemic encephalopathy	Gen	N	21	76	Gov	Y	3088	191	168	157	12 Parents not approached (12 "consent not requested")<br>7 Parents declined<br>3 Clinician refused<br>1 Clinical reason (1 ECMO)<br>11 Other (11 incomplete follow-up or lost to follow-up)	82%	0.1
Makker, 2020	RCT comparing rates of initial extubation success in preterm infants extubated to NIPPV versus NI-NAVA	Gen	N	1	NR	NR	Y	NR	59	30	26	27 Parents not approached (27 "not approached for consent")<br>2 Parents declined<br>4 Clinical reason (4 patients who were clinically unstable and therefore did not receive the assigned intervention)	44%	

© 2024 Shaikh H et al. *JAMA Network Open.*

Marr, 2019[1]	RCT to evaluate the impact of a 42-day compared to a 9-day course of dexamethasone given between 10-21 days of life on intact survival at 7 years of life (normal neurologic exam, IQ >70, attending school without supplemental educational support) among infants born at 24-27 weeks gestational age meeting respiratory criteria for evolving BPD	Gen	N	1	50	NR	Y	371	73	59	54	14 Parents declined<br>3 Death<br>2 Other (2 loss to follow-up)	74%	1.1
McMichael, 2021	Pilot RCT evaluating transfusion of fresh frozen plasma every 48 hours compared to usual care for frequency of ECMO circuit change	Surg	N	1	28	Gov, Hosp	Y	54	46	31	31	14 Parents declined<br>1 Clinician refused	67%	1.1
Mu, 2021	RCT evaluating the effect of drawing admission labs from umbilical cord versus the infant on hemoglobin concentration at 24 hours of life among VLBW infants	Gen	Y	3	68	None	Y	NR	98	80	80	3 Study factor (3 "unexpected eligibility")<br>9 Parents not approached (9 "not notified/approached")<br>2 Parents declined<br>4 Shortened consent window (4 "precipitous delivery")	82%	0.4

© 2024 Shaikh H et al. *JAMA Network Open.*

Napolitano, 2021	RCT evaluating the tolerability and efficacy of two different dose levels of albuterol for improving expiratory flow among ventilated preterm infants with severe bronchopulmonary dysplasia	Gen	U	1	23	Ind	Y	133	40	25	24	15 Parents declined<br>1 Consent withdrawn (by clinician)	60%	1.0
Nelson, 2021	RCT evaluating efficacy of topical mupirocin compared to placebo for reducing *Staphylococcus aureus* colonization (measured via a negative PCR screen 2 weeks after treatment)	Gen	N	1	39	NR	Y	2689	301	216	205	11 Study factor (11 "hospital discharge before first repeat Staphylococcus aureus screen")<br>85 Parents declined	68%	5.3
Niebler, 2021	RCT comparing platelet counts among infants who had nitric oxide versus placebo added to oxygenator sweep gas during cardiopulmonary bypass	Surg	N	1	NR	Ind	N	NR	61	40	40	1 Parents not approached (1 "taken to surgery before the study team had a chance to approach for consent")<br>20 Parents not approached/declined	66%	
Pandey, 2020	RCT evaluating lactose-free to lactose-containing formula for impact on cumulative morphine dose required among infants being treated for neonatal abstinence syndrome	Gen	U	1	43	NR	Y	NR	319	74	69	1 Study factor (1 "enrolled in error")<br>185 Parents not approached (185 eligible patients not approached for consent)<br>60 Parents declined<br>4 Consent withdrawn (not specified by whom)	22%	1.6

© 2024 Shaikh H et al. *JAMA Network Open.*

Parker, 2019	RCT to evaluate the impact of omitting compared to performing gastric residual volume measurements on weekly enteral nutrition volume over the 6-week study period among infants born at <= 32 weeks gestational age with a birth weight <1250g and receiving feeds within 72 hours after birth	Gen	N	1	36	Gov	Y	NR	308	146	143	13 Not approached (13 “parents or research team members unavailable to consent”)<br>76 Parents declined<br>56 Participating in another study<br>2 Consent withdrawn (by parent)<br>33 Clinical reason (32 “not expected to survive,” 1 clinical change such that subject no longer met inclusion criteria)<br>5 Other (5 “mothers too unstable to consent”)	46%	4.0
Perretta, 2020[1]	Randomized study comparing delayed cord clamping for 30 versus 60 seconds in preterm infants for impact on initial hematocrit measurement	DR	Y	1	12	NR	Y	577	374	105	105	269 Parents not approached/declined to participate (269 “declined enrollment or missed approach for consent”)	28%	8.8
Petrikin, 2018[1]	RCT comparing differential rates of attaining a genetic diagnosis within 28 days from testing between infants receiving rapid whole genome sequencing plus standard genetic tests compared to standard genetic tests alone	Gene	U	1	21	Gov	Y	129	111	65	65	11 Study factor (11 patients with “incomplete nomination”)<br>17 Parents not approached (9 patients who were discharged prior to enrollment, 8 “exceeded maximum parental consent attempts”)<br>17 Parents declined<br>1 Clinician refused	59%	3.1

© 2024 Shaikh H et al. *JAMA Network Open.*

Phelps, 2018[1]	RCT comparing effect of *myo*-inositol to placebo on outcomes of ROP requiring surgical intervention or death	Gen	N	18	17	Gov, Ind	Y	1398	1323	638	633	601 Parents declined<br>5 Death<br>84 Other (83 “other,” 1 “provided consent but not randomized”)	48%	2.1
Pickler, 2020	Randomized trial comparing touch or holding during feeding to standard feeding care among preterm infants for impact on time to full oral feeding	Gen	N	2	39	Gov	N	NR	NR	120	91	29 Other (29 unaccounted for)		1.2
Pineda, 2021	RCT comparing exposure to age-appropriate sensory stimulation versus standard care for impact on neurodevelopment measured as Ages and Stages Questionnaire score at 1 year corrected age among preterm infants	Gen	N	1	11	Gov, Priv	Y	925	102	70	52	6 Parents not approached (6 “did not approach”)<br>22 Parents declined<br>5 Consent withdrawn (not specified by whom)<br>10 Clinical reason (10 transferred to another facility)<br>7 Death	51%	4.7
Poola, 2019	RCT to evaluate the impact of primary closure compared to bedside silo and delayed closure on length of hospital stay among infants born at >= 34 weeks gestational age with gastroschisis	Surg	Y	1	60	None	Y	71	60	46	38	14 Parents declined/not approached<br>7 Clinical reason<br>1 Death	63%	0.6

© 2024 Shaikh H et al. *JAMA Network Open.*

Pourmogha-dam, 2020	Randomized trial comparing placement of a prophylactic passive peritoneal drain to no placement among infants requiring cardiopulmonary bypass for surgical correction of congenital heart disease for impact on time to negative total fluid balance	Surg	U	1	27	Hosp, Priv	Y	NR	27	26	25	1 Study factor (1 “did not receive allocated intervention”)<br>1 Clinical reason	93%	0.9
Ramanathan, 2020	RCT evaluating CHF5633 synthetic surfactant versus poractant alfa for impact on oxygen requirement, respiratory severity scores, development of BPD, and death at 36 weeks postmenstrual age in preterm newborns with respiratory distress syndrome	Gen	N	17	27	Ind	Y	297	126	123	113	3 Consent withdrawn (by parent)<br>10 Clinical reason	90%	0.2
Roberts, 2018[1]	RCT comparing the effects of surfactant delivery via a laryngeal mask airway followed by CPAP to CPAP alone on the need for intubation and mechanical ventilation in the first 7 days of life	Gen	N	7	51	NR	Y	3594	1705	103	103	2 Parents not approached (2 "unable to consent")<br>19 Parents declined to participate<br>18 Clinical reason	6%	0.3
Romano-Keeler, 2017	RCT to determine the impact of oral colostrum priming compared to no oral priming on salivary immune peptides and oral microbiota	Gen	U	1	18	Gov, Priv	Y	1885	370	99	99	29 Parents declined<br>242 Other (237 "Other (competing studies, Spanish speaking only)," 5 unaccounted for)	27%	5.5

© 2024 Shaikh H et al. *JAMA Network Open.*

Rosenfeld, 2021	RCT evaluating safety and efficacy of tin mesoporphyrin compared to placebo for reducing total serum bilirubin among infants receiving phototherapy for hyperbilirubinemia related to hemolytic disease	Gen	N	18	30	NR	N	NR	NR	91	91	N/A		0.2
Rosterman, 2018	Randomized crossover trial assessing the effect of neurally adjusted ventilatory assist compared to synchronized intermittent mandatory ventilation on respiratory severity scores	Gen	N	1	21	Hosp	Y	149	70	24	22	26 Study factor (25 "consenter unavailable," 1 "randomized but data were not collected in entirety")<br>3 Parents not approached ("3 parent unavailable")<br>11 Parents declined<br>3 Clinician refused<br>4 Participating in another study<br>1 Clinical reason	31%	1.0
Ruoss, 2021[2]	Pilot RCT evaluating withholding vs administering antibiotics immediately after birth for preterm infants for effect on composite outcome of late onset sepsis, bronchopulmonary dysplasia, necrotizing enterocolitis, and death	Gen	Y	1	25	Gov	Y	197	156	98	98	4 Study factor (4 "study team not called after birth")<br>50 Parents declined<br>3 Clinician refused<br>1 Other (1 "Spanish speaking only)	63%	3.9

© 2024 Shaikh H et al. *JAMA Network Open.*

Salas, 2020	RCT evaluating percent body fat at 3 months corrected age among preterm infants who had versus did not have body composition reports made available to their treating physicians	Gen	N	1	NR	Gov	Y	105	102	50	25	39 Parents declined<br>2 Participating in another study<br>36 Other (11 "other," 25 loss to follow-up)	25%	
Salas, Jerome, Finck, et al., 2021[1]	RCT comparing supplemental protein included in fortified human milk feeds versus a standard level of protein for effect on % body fat at 30-32 weeks postmenstrual age and 3 months corrected age among preterm infants	Gen	N	1	18	Gov, Hosp	Y	141	138	56	50	6 Study factor (6 "missing data")<br>56 Parents declined<br>11 Clinical reason (11 died or had SIP/NEC)<br>15 Other	36%	2.8
Salas, Li, Parks, et al., 2018	RCT examining feasibility of early (starting on day of life 1) compared to delayed (starting on day of life 5) feeding as well as efficacy related to number of full enteral feeding days in the first month of life among extremely preterm infants	Gen	N	1	10	Gov	Y	114	105	60	60	26 Parents declined<br>19 Other	57%	6
Salas, Woodfin, Phillips, et al., 2018	RCT comparing two vitamin D doses and placebo for effect on Bayley III cognitive score at 22-26 months of age	Gen	N	1	NR	Gov, Hosp	Y	307	236	100	70	91 Parents declined<br>20 Death<br>55 Other (45 "other," 10 loss to follow-up)	30%	

© 2024 Shaikh H et al. *JAMA Network Open.*

Schanler, 2018	RCT comparing the effects of acidified verses non-acidified liquid human milk fortifier on weight gain	Gen	N	14	29	Ind	Y	1354	382	164	160	4 Study factor (4 "never received study fortifier")<br>218 Parents declined	42%	0.4
Sekar, 2019[1]	RCT to evaluate the impact of delivery room resuscitation with inhaled nitric oxide compared to placebo on total exposure to supplemental oxygen as well as heart rate, oxygen saturation, and need for intubation in the first 20 minutes of life among infants 25 0/7 - 31 6/7 weeks gestational age requiring PPV with supplemental oxygen at birth	DR	Y	1	60	Ind	Y	NR	171	34	28	2 Study factor (2 "no data capture")<br>59 Parents declined<br>25 Clinical reason<br>31 Consented prenatally and no longer eligible at time of allocation to intervention<br>26 Other (12 "equipment malfunction, no team available, other," 12 "non-qualifying exclusion," 2 "other")	16%	0.5
Shankaran, 2017	RCT comparing the effects of four combinations of therapeutic cooling depths and durations on death and neurodevelopmental outcomes among infants with hypoxic ischemic encephalopathy	Gen	N	18	64	Gov	Y	1261	514	364	347	45 Parents not approached (45 "consent not requested")<br>100 Parents declined<br>5 Clinician refused<br>17 Other (17 loss to follow-up)	68%	0.3

© 2024 Shaikh H et al. *JAMA Network Open.*

Shankaran, 2018	RCT comparing the effect of weaning from the incubator at 1600g verses 1800g on length of hospital stay among infants born at 29-33 weeks gestational age with birth weight <1600g	Gen	N	17	14	Gov	Y	1565	885	366	363	106 Parents not approached (66 "parent not available," 40 "parent not approached")<br>364 Parents declined<br>8 Clinician refused<br>5 Consent withdrawn (4 by parent and 1 by clinician)<br>3 Clinical reason<br>36 Other	41%	1.5
Shellaas, 2019	Prospective trial to evaluate the impact of exposure to mother's voice (recorded book reading) compared to standard NICU ambient noise on sleep structure and the probability of awakening among infants born at 33-41 weeks gestational age	Gen	N	1	NR	Gov, Priv	N	NR	NR	47	47	N/A		
Shirk, 2019	RCT to evaluate the impact of umbilical cord milking compared to delayed cord clamping on initial hematocrit concentration among infants born at 23 to 34 weeks gestational age	DR	Y	1	49	NR	Y	4296	307	282	204	25 Parents declined<br>78 Consented prenatally and no longer eligible at time of allocation to intervention	66%	4.2

© 2024 Shaikh H et al. *JAMA Network Open.*

Smith, 2018	Trial evaluating non-inferiority of infrared temporal artery thermometer readings compared to axillary temperature readings in a cohort of preterm neonates between 28 to 36 weeks postmenstrual age	Gen	N	1	NR	Hosp	N	NR	NR	68	68	N/A		
Song, 2019	RCT to evaluate the impact of a pulsatile pacifier (NTrainer system) compared to standard, non-pulsatile pacifier on time to full oral feeds among infants born at 26-30 weeks gestational age	Gen	N	5	48*	Ind	Y	377	357	210	197	34 Parents not approached (34 "not approached")<br>18 Parents declined<br>45 Participating in another study<br>35 Clinical reason (28 "clinical change," 7 transferred to another facility)<br>1 Death<br>27 Other (27 "other non-medical reasons")	55%	0.8
Sood, 2021[1]	Randomized trial evaluating feasibility, safety, and efficacy for reducing need for intubation of four dosing schedules of aerosolized surfactant using two nebulizer devices in preterm infants with respiratory distress syndrome	Gen	N	1	56	Gov	Y	702	262	159	149	10 Parents not approached (6 "outside window for eligibility," 4 "relative unavailable")<br>86 Parents declined<br>13 Clinical reason<br>2 Consented prenatally and no longer eligible at time of allocation to intervention<br>2 Other (1 "did not speak English," 1 "baby up for adoption")	57%	2.7

© 2024 Shaikh H et al. *JAMA Network Open.*

Soul, 2021[1]	RCT of bumetanide added to phenobarbital vs. phenobarbital alone for treating neonatal seizures	Gen	N	4	NR	Gov, Hosp, Priv	Y	539	273	111	43	86 Study factor (82 "other, e.g., logistical issues," 4 "seizures missed on EEG)<br>80 Parents declined<br>64 Clinical reason	16%	
Stokes, 2018	Trial evaluating the effect of exposure to music on sleep-wake cycles in late preterm neonates	Gen	N	1	NR	None	Y	129	100	34	30	4 Study factor (4 "excluded due to artifact")<br>47 Not approached (47 “missed”)<br>17 Parents declined<br>2 Other (2 unaccounted for)	30%	
Suterwala, 2017	Trial evaluating the safety and reliability of fiberoptic endoscopic evaluation of swallowing and videofluoroscopic swallowing study among infants younger than 3 months of age	Gen	N	1	10	Hosp	N	NR	NR	25	25	N/A		2.5
Travers, 2018	Trial comparing the effect of environmental oxygen delivery via servo-controlled incubator versus nasal cannula on episodes of intermittent hypoxemia among preterm neonates	Gen	N	1	6	Gov, Hosp	Y	63	49	27	25	19 Parents not approached (19 "parent/guardian not available")<br>3 Parents declined<br>1 Consent withdrawn (by parent)<br>1 Clinical reason	51%	4.2

© 2024 Shaikh H et al. *JAMA Network Open.*

Travers, 2020	RCT evaluating higher volume (180-220 ml/kg/d) to usual-volume (140-160 ml/kg/d) feeds for impact on growth velocity among infants with birth weight 1001-2500g	Gen	N	1	42	Hosp	Y	NR	416	224	217	89 Parents not approached (89 "[parent] not available")<br>103 Parents declined<br>7 Consent withdrawn (by parent)	52%	5.2
Viscardi, 2020[1]	RCT evaluating intravenous azithromycin compared to placebo for outcome of survival to discharge with 3 negative *Ureaplasma* tracheal aspirate cultures	Gen	N	7	27	Gov	Y	982	434	121	121	89 Not approached (89 "missed")<br>194 Parents declined<br>30 Other (23 "non-English speaking parent," 7 "maternal age <18 years")	28%	0.6
Vittner, 2018	Trial evaluating the impact of skin-to-skin care on infant and parent salivary oxytocin and cortisol levels as well as parental anxiety and observed parent-infant synchrony and responsiveness among stable preterm infants born at 30-34 weeks	Gen	N	3	NR	Priv, Hosp	N	112	58	28	28	4 Study factor (4 "discharged prior to data collection")<br>26 Parents declined	48%	
Wallace, 2017	De-escalation trial to identify whether lower-dose bevacizumab injections could successfully treat retinopathy of prematurity	Gen	U	NR	17	Gov	N	NR	NR	61	58	3 Other (3 unaccounted for)		

© 2024 Shaikh H et al. *JAMA Network Open.*

Wallace, 2020	Dose-escalation study with objective of determining the lowest effective dose of intravitreous bevacizumab for treating retinopathy of prematurity	Gen	U	11	26	Gov	N	NR	NR	59	55	4 Other (4 unaccounted for)		0.2
Willeitner, 2017	RCT comparing the effects of the concentrated preterm formula Similac Special Care 30 with iron to powdered Similac Human Milk Fortifier on weight gain among very low birth weight neonates	Gen	U	1	NR	NR	Y	NR	NR	85	70	2 Study factor (2 "incomplete data")<br>1 Consent withdrawn (not specified by whom)<br>12 Clinical reason		
Zuzarte, 2017	Trial evaluating the impact of stochastic vibrotactile stimulation on hyperirritability among neonates receiving pharmacologic treatment for neonatal opioid withdrawal syndrome	Gen	U	1	NR	Gov, Hosp	N	NR	NR	26	26	N/A		

Table notes:
Abbreviations: DR = delivery room, Gen = general NICU, Surg = surgical, Gene = genetics, Y = yes, N = no, U = unclear, Gov = governmental funding, e.g., NIH, Hosp = hospital funding, Ind = industry funding, Priv = private organization funding, NR = not reported, Proportion of eligible patients included in results = N in results/N eligible, Participant accrual rate = included participants/sites (N)/enrollment duration (months)

Reasons for non-inclusion: the specific reason is provided along with the number of relevant patients for cases in which categorization relied on some interpretation; unless otherwise stated, "clinical reason" refers to a change in the patient's medical status such that they no longer met inclusion criteria or were too ill to be included in the study; "other" refers to "other" explicitly provided as the reason in the article unless a more specific reason is provided in the table; "shortened window for consent" was combined with "parents not approached" for our analysis given the small number of patients in this category and overlap between these categories.

[1]Study ended earlier than planned

© 2024 Shaikh H et al. *JAMA Network Open.*

[2]Study was paused for a full IRB review due to a high number of adverse outcomes and was then resumed given that the incidence of adverse events was similar to the national standard as demonstrated by Vermont Oxford Network data

Article-specific comments:
Angeles, 2020: The article reported that 100% of eligible infants were enrolled, which is notable because it may reflect recruitment practices that are worth examining and emulating.
Butler-O'Hara, 2017: There was a discrepancy in numerical descriptions provided in the text compared to the flow diagram: while the flowchart describes that 312 patients were assessed for eligibility and 83 of these were "missed," the text describes that 162 patients for whom there was "inability to obtain consent before the neonate started phototherapy" (study factor). We opted included 162 patients under study factor and adjusted the number of patients screened to 391.
Ceyhan-Birsoy, 2019: A flow diagram was not included in this article but the article did reference a flow diagram from a previously published article.
Cooper, 2020: There was a discrepancy in the article's reporting such that the difference between the reported numbers of eligible patients and those included in the analysis was 2 fewer than the summed total of reasons provided for non-inclusion of patients.
DuPont, 2021: There were some discrepancies between reporting in the article text and the flow diagram. While the flow diagram described that 28 patients were eligible for inclusion, the text states that 29 were. In addition, while the flow diagram described that parents of 7 patients declined to participate, the text indicates this was the case for 8 patients. We opted to use the numbers in the text in our analysis (29 eligible patients and 8 for whom parents declined), as these were also supported by percentage descriptions given in the text.
Gray, 2017: There was a discrepancy in the article's reporting such that the difference between the reported numbers of eligible patients and those included in the analysis was 1 fewer than the summed total of reasons provided for non-inclusion of patients in the text.
Hammond, 2021: Though we used 178 as the reported number of eligible patients, the actual number is likely higher, as the article states "178 infants and their families were approached for consent."
Katheria, 2017: This trial recruited from a pool of patients who were simultaneously recruited for an observational study of EEG use during delivery room resuscitation. Since 200 patients were screened for the EEG study and 197 deemed to be eligible, these were the numbers we used in our analysis.
Kim, 2020: This article only reported the numbers of patients who were eligible and whose parents consented. They report that 35 mothers, 5 of whom had twins, consented to their infants' participation, giving 40 eligible patients. However, it is likely that more patients were eligible that did not consent.
Makker, 2020: This article reported that 59 patients were screened, however, based on the subsequent reasons for non-inclusion provided (reasons provided were "not approached" and "declined" with no mention of not meeting inclusion criteria), this seemed to actually be the number eligible and was included as such in our analysis.
Parker, 2019: There was a discrepancy in this article's reporting such that the difference between the reported numbers of eligible patients and those included in the analysis was 20 fewer than the summed total of reasons provided for non-inclusion of patients.
Pourmoghadam, 2020: This article stated that, "informed written consent was obtained from 27 parents or legal guardians." We used 27 as the number of patients eligible, though the true number of eligible patients was not reported.
Roberts, 2018: Though we used 1705 as the number of eligible patients, it is not clear that this is accurate. The article states that 1705 patients are on CPAP, though only 142 patients were approached. Because the inclusion and exclusion criteria were written such that not all infants on CPAP were eligible, it is likely that the true number of eligible patients was less than 1705 and greater than 142.

© 2024 Shaikh H et al. *JAMA Network Open.*

Shirk, 2019: The true number of eligible patients was not clearly reported in the article; according to the flow diagram in the article, 282 patients would have been eligible, however, the flow diagram also indicates that parents of an additional 25 patients not included among these 282 were approached for consent but declined. Because we presume that parents of patients who were approached for consent were necessarily eligible for participation, we estimated that at least 307 (282 + 25) patients were eligible.
Travers, 2020: This article uses the language, “416 infants screened,” however, subsequent reasons for non-inclusion of screened patients include not approached, declined, and consent withdrawn with no mention of patients who did not meet eligibility criteria. We therefore inferred that this was actually the number of patients eligible for participation.
Wallace, 2017: The article refers to “all participating institutions,” implying that the study took place at multiple sites, however, it does not clearly state how many sites were included.

© 2024 Shaikh H et al. *JAMA Network Open.*
