# Supplementary material for: Eligible Infants Included in Neonatal Clinical Trials and Reasons for Noninclusion: A Systematic Review
Source: JAMA Netw Open. 2024 Oct 25;7(10):e2441372. doi: 10.1001/jamanetworkopen.2024.41372 (PMC11581680; doi:10.1001/jamanetworkopen.2024.41372)
Supplement: Supplement 2. — Data Sharing Statement [file jamanetwopen-e2441372-s002.pdf]

Data Sharing Statement

Shaikh. Eligible Neonates Included in Clinical Trials and Reasons for Noninclusion. *JAMA Netw Open*. Published October 25, 2024. doi:10.1001/jamanetworkopen.2024.41372

Data
Data available: No

Additional Information
Explanation for why data not available: no patient data
